# Sydenham Society of London

**Published:** 1850-09

**Authors:** 


### THE MEDICAL EXAMINER.

### PHILADELPHIA, SEPTEMBER, 1850.

### SYDENHAM SOCIETY OF LONDON.

In the pages of the Examiner we have drawn the attention of our readers from time to time to this valuable Association, which for seven years has been pursuing unobtrusively, but beneficially, the even tenor of its way. Its officers, constituted of the distinguished members of the profession, have, in the most disinterested manner, assumed the office of caterers for their medical brethren, and the Society has every year issued in a uniform and elegant style, and at a comparatively trifling rate, books of the highest order of merit. Desirous, however, of extending their usefulness, the Society in their Report of the seventh general meeting, makes the following appeal, which we fervently hope may be responded to by the numerous members.

"In the Fifth Annual Report it was announced that arrangements were pending for the publication of a British Medical Bibliography, and the Council have now to announce that they have agreed with Dr. Kennedy, late of Ashby-de-la-Zouch, to publish his MS. Bibliography. On this work Dr. Kennedy has been occupied during the greater part of a long life, and has spared neither expense nor labor to make it, what the Council believe it will prove to be, a most complete and valuable Bibliography of British Medicine. The Council have been anxious not to publish any portion of the Bibliography, till they had made such arrangements as would justify the expectation that they might ultimately carry into effect the whole scheme. Having, however, now arranged for the preparation of the general Biblography to the end of the sixteenth century, and for the whole of the British department to the close of the last century, it is the intention of the Council to issue at least one volume in the course of the ensuing year. It is deemed highly desirable that the issue of Bibliography should interfere as little as possible with that of the ordinary works. But in order to accomplish this object, it will be absolutely necessary to keep up, if not to augment, the present number of Members ; for, in consequence of the number of volumes that have been issued for each year's Subscription, the Council have been unable to set aside any portion of the funds for the special purposes of the Bibliography, although, as it was originally intended, this they have been anxious to do. The Council therefore trust that all who have benefitted by the prosperity which the Society has hitherto enjoyed, will feel it incumbent on them to seek every opportunity of promoting its future success by inducing others to participate in its advantages."

